# Role of Microglia Adenosine A_2A_ Receptors in Retinal and Brain Neurodegenerative Diseases

**DOI:** 10.1155/2014/465694

**Published:** 2014-07-16

**Authors:** Ana R. Santiago, Filipa I. Baptista, Paulo F. Santos, Gonçalo Cristóvão, António F. Ambrósio, Rodrigo A. Cunha, Catarina A. Gomes

**Affiliations:** ^1^Centre of Ophthalmology and Vision Sciences, IBILI, Faculty of Medicine, University of Coimbra, 3000-548 Coimbra, Portugal; ^2^AIBILI, 3000-548 Coimbra, Portugal; ^3^Center for Neuroscience and Cell Biology, Largo Marquês de Pombal, Universidade de Coimbra, 3004-517 Coimbra, Portugal; ^4^Faculty of Medicine, University of Coimbra, Azinhaga de Santa Comba, Celas, 3000-548 Coimbra, Portugal; ^5^Department of Life Sciences, Calçada Martim de Freitas, 3000-456 Coimbra, Portugal

## Abstract

Neuroinflammation mediated by microglial cells in the brain has been commonly associated with neurodegenerative diseases. Whether this microglia-mediated neuroinflammation is cause or consequence of neurodegeneration is still a matter of controversy. However, it is unequivocal that chronic neuroinflammation plays a role in disease progression and halting that process represents a potential therapeutic strategy. The neuromodulator adenosine emerges as a promising targeting candidate based on its ability to regulate microglial proliferation, chemotaxis, and reactivity through the activation of its G protein coupled A_2A_ receptor (A_2A_R). This is in striking agreement with the ability of A_2A_R blockade to control several brain diseases. Retinal degenerative diseases have been also associated with microglia-mediated neuroinflammation, but the role of A_2A_R has been scarcely explored. This review aims to compare inflammatory features of Parkinson's and Alzheimer's diseases with glaucoma and diabetic retinopathy, discussing the therapeutic potential of A_2A_R in these degenerative conditions.

## 1. Introduction

### 1.1. Role of Microglia in Brain Physiology

In the central nervous system (CNS), microglial cells participate in innate immunity; microglia can respond to different types of signals, namely the presence of pathogens (extrinsic signals) or to intrinsic signals, namely diffusible mediators released by stressed neurons, astrocytes or microglia (reviewed in [1]). Although the present review mainly focuses on the contribution of microglia to the pathophysiology of neurodegeneration in the brain and the retina, any attempt to interfere with microglia in pathological conditions also needs to take into account the role of microglia in physiological conditions.

In the healthy brain, the majority of microglial cells exhibit a ramified phenotype, compatible with a surveillance function of the surrounding environment. This crucial sensor ability is supported by the constant extension and retraction of cellular processes [2, 3]. This dynamics is not random but instead instructed by increased neuronal activity, that activates pannexin-1 hemichannels, triggering the diffusion of signals, namely, ATP, that drive process motility towards that specific neuron [4]. The interconversion between the so-called “surveying” phenotype (considered more adequate, as compared to the old terminology “resting” phenotype) and the “alerted” phenotype can be driven either by external stimuli (e.g., pathogens) or by neural signals. The latter is achieved by direct neuron-microglia contact or by diffusible mediators (reviewed, e.g., in [1]). This activation of microglia drives some immediate responses that mainly consist in (1) production/release of rectifier mediators and (2) phagocytosis of neurons or subcellular components (mainly dendritic spines and synapses). Microglial phagocytosis of neurons or neuronal structures has been mostly studied in pathological conditions (e.g., [5–8]), but it also takes place in nonpathological conditions. In fact, it is a process of particular importance during neurodevelopment, as shown by Tremblay and coworkers [9] in the visual system: light deprivation and the subsequent decrease in the workload of neuronal circuits involved in visual processing lead to the engulfment of synaptic elements by microglia. This physiological process, termed synaptic pruning, is regulated by the immune system; synapses and axons to be phagocytosed are labeled by the complement components C1q and C3, which prompt their selective recognition by microglial cells [10–12]. Synaptic pruning is crucial to normal brain wiring and function and any impairment of this process may impact on neurodevelopment. For instance, this was recently associated with deficits in synaptic transmission, which are paralleled by behavioral abnormalities characteristic of disorders of the autism spectrum and other neuropsychiatric conditions [13]. This process also occurs during adulthood, particularly in neurogenic niches of the brain, such as the hippocampus, where microglia phagocytose apoptotic newborn neurons [14].

Intriguingly, as part of their physiological role, microglia also actively shape their neuronal environment thanks to their ability to trigger neuronal death [15–17]. Again, such a role has a particular relevance during brain development, namely, during the first postnatal week, as heralded by the observation that microglia accumulate in regions of developmental cell death in the embryonic cerebral cortex [18]; furthermore, in the spinal cord, the cell death of motor neurons correlates temporally with the arrival of microglia [19].

In addition to their role in synaptic pruning, microglia also regulate synapse formation [20–22]. This function has been shown to be dependent on the production and release of mediators, such as brain-derived neurotrophic factor [20] or interleukin- (IL-) 10 [22], although other diffusible mediators are likely to be involved. This critical function of microglia must be strictly preserved in order to prevent neurodevelopmental deficits, as suggested by a recent* in vitro* study showing that activation of microglia by an inflammatory stimulus may impact on the presynaptic differentiation of immature neurons [23].

Microglial support to synapse formation/elimination is tightly associated with the newly recognized role of microglia as active partners in the transmission of information within synapses [24]. Thus, recent studies show that microglia also monitor the functional state of synapses and respond to changes in synaptic activity [25, 26]. Accordingly, the highly motile processes of microglia contact with synapses and regulate synaptic transmission in nonpathological conditions [9, 10, 27–30].

### 1.2. Role of Microglia in Retinal Physiology

**Figure 1:**
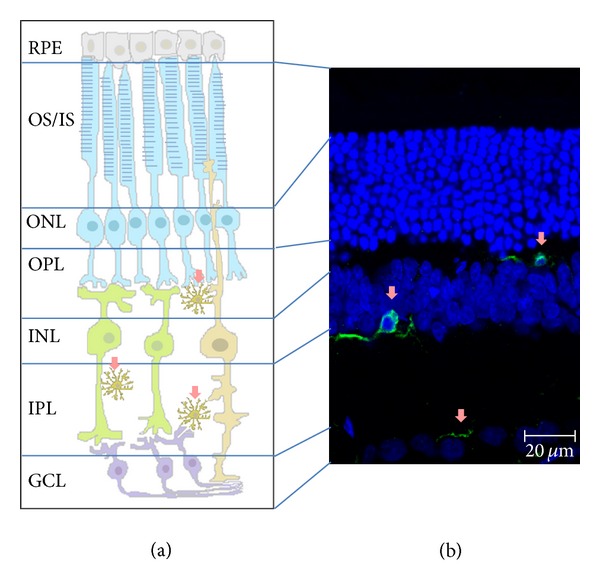
Microglial localization in the retina. Microglial cells in a “surveying” state (pink arrows) in nonpathological conditions are mainly located in the plexiform layers. Retinal layers: OS/IS, outer and inner segments of rods and cones; ONL, outer nuclear layer; OPL, outer plexiform layer; INL, inner nuclear layer; IPL, inner plexiform layer; GCL, ganglion cell layer. Schematic draw of the retinal layers (a) and confocal image from a retinal section where the different layers are depicted (b): nuclear layers (in blue) and microglia cells (in green).

In the adult retina, the presence of microglia has been described in several mammals species, including rabbits [31–33], mice [34], rats [31, 35, 36], monkeys [37, 38], and humans [39–41]. Microglial cells in the adult normal retina are mainly located in the inner vascularized regions, that is, the nerve fiber and ganglion cell layers and in plexiform layers, whereas they are scarce in the inner nuclear layer and absent in the outer nuclear layer (Figure 1).

In the healthy retina, microglial cells represent a self-renewing population of innate immune cells, which constantly survey their microenvironment, as occurs in the brain. Retinal microglia can also phagocytose pyknotic cells generated upon neural remodeling of the retina [42]. A more recent study performed in zebrafish showed that microglial cells not only have a “cleaning” role in the developing retina, but also are required for normal retinal growth and neurogenesis [43]. Microglia may also play a role in the formation of blood vessels in the developing retina, since microglia depletion during retinal development reduces vascularization, an effect restored by intravitreal injection of microglia [44]. This is in agreement with the origin of retinal microglial cells that originate from cells of mesodermal lineage [45] and populate the retina before vascularization and along with the onset of vasculogenesis [46].

### 1.3. A_2A_R Regulation of Microglia Physiology

Adenosine is a neuromodulator, which also exerts important functions in the immune-inflammatory system [47]. Microglial cells express all subtypes of adenosine receptors, A_1_, A_2A_, A_2B_, and A_3_ receptors [48]. Although a large body of evidence highlights the ability of A_1_ and A_3_ receptors to regulate microglia responses, such as proliferation, morphological phenotype, and release of mediators [49–52], particular attention has been paid to A_2A_R, considered to have a central role in the pathophysiology of degeneration [53–55].

It is claimed that A_2A_R modulation (both activation and blockade) interferes with microglia-mediated inflammation in degenerative conditions (see below). Of note, in physiological conditions, important functions operated by microglia, namely, the release of mediators, such as trophic factors [56] or nitric oxide (NO) [57], as well as the extension and retraction of processes that govern the surveying activity of microglia [58], are apparently out of A_2A_R control, until a pathologic insult triggers a gain-of-function of A_2A_R [56, 57, 59, 60]. However, the milestone study by Davalos et al. [2] shows that the baseline motility of microglial processes in the healthy brain is governed by ATP (and prevented by ATP degradation), as occurs in pathological-like conditions. This observation raises the unanswered question whether the activation of A_2A_R by ATP-derived adenosine regulates the dynamics of microglial processes in physiological conditions.

### 1.4. Role of Microglia in Degenerative Conditions of the Brain

**Figure 2:**
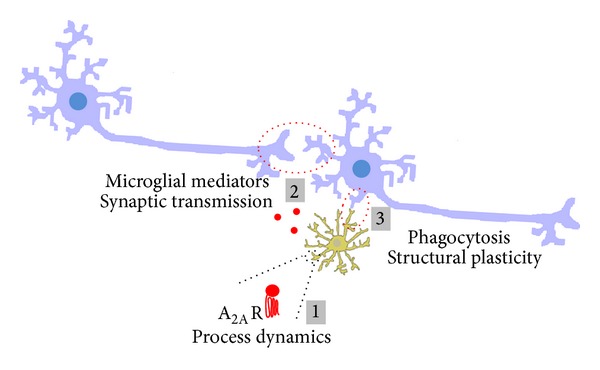
Microglia in the healthy brain/retina. Schematic representation of the main functions exerted by microglia (in yellow) under physiological conditions: surveying the environment by constant extension and retraction of processes (it remains to clarify if A_2A_R regulate this process, as occurs in pathology) (1); regulation of basal synaptic transmission and plasticity through the release of mediators (red circles), some of them being also important mediators of inflammation (2); regulation of spine/synapse structural plasticity, mainly by phagocytosis, a process regulated by inflammatory mediators, according to the neuronal workload (3).

The main physiologic roles operated by microglia (release of mediators that control synaptic transmission, synapse formation, and phagocytosis of cells or cellular elements) are strictly dependent upon their sensor ability. Any interference at this functional level may create conditions favoring the development of degenerative processes, which are bolstered by abnormal synaptic transmission, aberrant synapse formation and/or elimination, and abnormal phagocytosis (Figure 2). Therefore, the identification of molecular systems able to modulate microglial functions may help defining new pharmacological targets to interfere with the progression of neurodegenerative diseases. Indeed, microglia-driven neuroinflammation is associated with a broad spectrum of neurodegenerative diseases and has been more detailed in Alzheimer's disease (AD) and Parkinson's disease (PD).

The accumulation of misfolded *β*-amyloid-containing proteins (Abeta) and alpha-synuclein are histopathological hallmarks of established AD and PD, respectively [61–67]. Protein aggregates can directly exert neurotoxicity [68–70] and can trigger parallel maladaptive changes of glial cells; in fact, animal models of AD and PD and postmortem examination of the brain of AD or PD patients frequently reveal increased numbers of activated microglia in degenerated brain regions [71–76]. Moreover,* in vivo* studies using PET with a radiotracer for activated microglia in AD and PD patients have provided evidence for increased levels of activated microglia in brain regions that are affected by the disease [75–79]. Importantly, protein aggregates may be sufficient causative factors for microglial activation and release of inflammatory mediators [80], which, in turn, amplify neuroinflammation and further exacerbate neurodegeneration [73]. Such a scenario prompts the idea that microglia-induced neuroinflammation may play a critical role in the progression of neurodegenerative conditions [65–67, 81, 82].

Indeed, several microglia-derived inflammatory mediators have been shown to be involved in neuronal damage in neurodegenerative diseases. Thus, one possible causative factor for neuronal death in AD is A*β*-induced NO production by microglia [83]. Furthermore, A*β* and interferon-gamma (IFN-*γ*) can activate microglia to produce reactive nitrogen intermediates and tumor necrosis factor (TNF), contributing to neuronal degeneration observed in AD [84]. Additional proof-of-concept for the role of microglia in the progression of neuronal damage in AD was derived from the observation that drugs preventing microglial activation indeed delay the emergence of an AD-like phenotype in animal models [85]. Similarly, increased expression of inflammatory mediators is also found in PD animal models [51, 80, 86] and in postmortem PD brains [87, 88], including proinflammatory cytokines, such as IFN-*γ*, IL-1*β*, TNF, IL-2, and IL-6, released by microglia [89–91]. The microglial overactivation and the release of proinflammatory cytokines and reactive oxygen species (ROS) are associated with neuronal loss in PD [72, 73]; further evidence for the key role of these microglia-derived mediators in the evolution of neuronal damage in PD was obtained by showing that the inactivation of microglia-derived mediators counteracts neurodegeneration in the MPTP (1-methyl-4-phenyl-1,2,3,6-tetrahydropyridine) animal model of PD [92–95].

In addition to the direct neurotoxic impact of these microglia-derived inflammatory mediators, the deregulation of the phagocytic activity of microglia also contributes to the progression of neuronal damage. This is heralded by the observations of an increased number of phagocytic microglia close to damaged neurons in PD [96, 97]; furthermore, blocking microglial activation attenuates neurodegeneration, further supporting the role of microglia in the evolution of the pathological process [98]. Increased phagocytosis of neuronal elements seems to be a selective process since* in vitro* studies have suggested that microglia may paradoxically reduce its ability to degrade A*β*-containing aggregates, and their intracellular accumulation leads to dysfunctional/dystrophic microglia [99–101]. In animal models of AD it has been shown in late stages of cerebral amyloidosis that the phagocytic capacity of microglia is impaired [102], and this impairment was described to accelerate pathology progression [103].

In summary, microglial functions, from the release of inflammatory mediators to the ability to phagocytose, are deregulated in neurodegenerative diseases. This implies that the identification of regulatory systems able to rebalance microglial function may be of therapeutic interest to manage the progression of neurodegenerative diseases.

### 1.5. Control of Microglia-Driven Neuroinflammation by A_2A_R in Brain Diseases

The ability of adenosine and A_2A_R activation to control the activation of different inflammatory cell types has been consistently documented by different groups [47]. Likewise, several* in vitro* and* in vivo* studies clearly demonstrate that A_2A_R controls several facets of microglia dynamics [56–58, 104, 105], such as (1) the proliferation, (2) the levels of inflammatory enzymes such as cyclooxygenase-2, and (3) the synthesis and release of inflammatory mediators. Furthermore, studies carried out in several models of brain disorders have found that pharmacological blockade or genetic inactivation of A_2A_R affords a robust neuroprotection [53, 54], and increasing evidence suggests this neuroprotection involves the control of microglia-mediated neuroinflammation [54, 106, 107]. Furthermore, different brain insults triggering neuroinflammation also cause an upregulation of A_2A_R [56, 60], namely, in microglial cells [56, 57, 59, 108], which is in line with the described ability of cytokines to upregulate A_2A_R (reviewed by [53]). Finally, A_2A_R seem to have an additional ability to protect neurons from proinflammatory priming neurodegeneration [109, 110]. This has bolstered the interest to exploit A_2A_R as a promising pharmacological target to control the neuroinflammatory component of neurodegenerative diseases, allowing the slowdown of their evolution [47, 56, 106, 107].

The clinical interest of the adenosine modulation system in the control of memory dysfunction in AD first arose from epidemiological studies showing an inverse correlation between the consumption of moderate doses of caffeine (a nonselective adenosine receptor antagonist) and the deterioration of memory performance upon aging and AD [111]. This was in notable agreement with animal studies showing that the chronic consumption of caffeine reduces cognitive impairment and decreases A*β* levels in the brain of transgenic mouse models of AD [112–114], as well as in mice exposed to A*β* [104, 115], a purported causative factor of AD [64]. Animal studies were paramount to identify A_2A_R as the likely targets of caffeine [116], since the pharmacological or genetic blockade of A_2A_R mimics the neuroprotective effects of caffeine [104, 117]. In accordance with the involvement of neuroinflammatory features in AD, the exposure of rodents to lipopolysaccharide (LPS), which is present in the cell wall of gram-negative bacteria and used as a prototypical activator of microglia, triggers the activation of microglia, a proinflammatory status in the brain parenchyma, and deterioration of synaptic plasticity and memory performance [105]. Notably, this LPS-induced neuroinflammation can be prevented both by the caffeine [118] and by the selective blockade of A_2A_R [60], which abrogates the LPS-induced dampening of hippocampal synaptic plasticity, the purported neurophysiological basis of learning and memory [119]. Further supporting this role of microglial A_2A_R in AD, the analysis of postmortem human cortex from AD patients revealed an increased density of A_2A_R [60] that is more prominent in microglia [120].

As in AD, there is also solid evidence for a role of A_2A_R in the control of PD, as testified by the recent introduction of A_2A_R antagonists as coadjuvants in the management of PD [121]. Thus, A_2A_R antagonists improve PD symptoms in different rodent and primate models of the disease and also in PD patients enrolled in clinical trials (for a review see [122]). Besides the control of motor function, A_2A_R blockade also dampens microglial activation in the striatum [108] and* substantia nigra* [123] in animal models of PD. Furthermore, caffeine downregulates microglia-driven neuroinflammatory responses and decreases NO production in animal models of PD [124]. Although caffeine acts on both A_1_R and A_2A_R, the neuroprotective properties of caffeine in PD are mediated through A_2A_R blockade [125, 126]. In fact, caffeine consumption has been associated with lower risk of PD in several case-control and cohort studies [127–132]. Interestingly, the association between coffee consumption and PD is strongest among subjects that slowly metabolize caffeine and are homozygous carriers of the CYP1A2 polymorphisms, the gene encoding for cytochrome P450 1A2 [133] which is the main enzyme involved in the metabolism of caffeine.

A recent* ex vivo* study (brain slices from MPTP-treated mice modeling PD) showed that a selective A_2A_R antagonist restores the ability of microglia to respond to tissue damage [134]. This A_2A_R-mediated control of neuroinflammation is argued to be critical for the neuroprotection afforded by A_2A_R blockade in PD since the inhibition of microglial function has been shown to be sufficient to decrease the dopaminergic neurodegeneration characteristic of PD.

**Figure 3:**
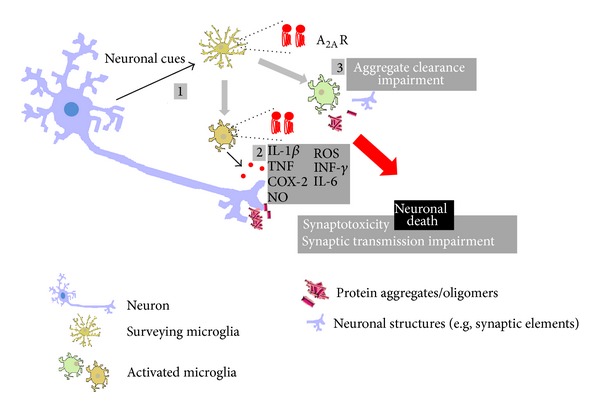
Microglia and neuroinflammation in the brain/retina. Schematic representation of the main inflammatory responses mediated by microglial cells (in yellow) in neurodegenerative conditions. Environment surveillance allows the detection of “pathological” events affecting neurons (in blue-purple); note that appropriate detection of danger signals may also be compromised under these conditions; one of the microglial changes consists in the upregulation of the expression/density of A_2A_R, as described in several degenerative disorders (1), usually paralleled by morphologic changes and by the release of inflammatory mediators (red circles), both anti- and proinflammatory molecules, that may impact on synaptic transmission, ultimately leading to synaptotoxicity (2); the ability of microglia to phagocytose subcellular components of damaged neurons or protein aggregates, typically present in some degenerative diseases, may also be impaired, further amplifying the cascade of events that lead to cell death/degeneration (3).

These two examples of neurodegenerative diseases support the working hypothesis that the beneficial effects resulting from A_2A_R blockade may involve their ability to attenuate microglial activation and associated chronic neuroinflammatory status, which would interrupt the vicious cross amplifying cycle of degeneration and inflammation leading to a slower development of neurodegenerative disorders (Figure 3).

### 1.6. Neuroinflammation Is a Common Feature between Retinal and Brain Degenerative Diseases

The combined effect of an ageing population and increasing life expectancy will increase the prevalence of chronic diseases [135], which encompass not only neurodegenerative brain diseases, but also retinal degenerative conditions amongst others. Indeed, the demographic evolution, with an increasing elderly population in western countries, exponentially augments the number of people at risk of age-related visual impairment caused by age-related retinal degenerative diseases [136]. Glaucoma and diabetic retinopathy are leading causes of blindness worldwide. Glaucoma is the second cause of irreversible blindness [137], affecting 70 million people worldwide and approximately 2% of the population over the age of 40 [138]. Diabetic retinopathy is a frequent complication of diabetes and may lead to blindness, making it one of the most feared complications of diabetes. Indeed, diabetic retinopathy is the leading cause of vision loss in working age adults [139]. Since the number of people affected by diabetes is expected to increase significantly in the next 25 years, from the actual 382 million to beyond 592 million [139], the number of people affected by diabetic retinopathy is expected to greatly expand.

The similarities between AD pathology and retinal degenerative diseases have been described elsewhere [140, 141], and neuroinflammation is a common feature between brain and retinal degenerative diseases. It is, thus, plausible to speculate that therapeutic agents and strategies used for brain neurodegeneration could also be considered for retinal diseases with an underlying chronic inflammation process. Retinal microglia cells express A_2A_R [142], opening the possibility that the control of microglia-mediated neuroinflammation through A_2A_R modulation might also be an attractive approach to manage retinal diseases.

### 1.7. Glaucoma Has a Neuroinflammatory Component

Glaucoma is defined as a group of ocular disorders of multifactorial etiology characterized by progressive optic neuropathy [143] and gradual loss of retinal ganglion cells and optic nerve (retinal ganglion cell axons) damage. Elevated intraocular pressure (IOP) is one of the major risk factors for developing glaucoma or glaucomatous neuropathy [144]. The current therapeutic approach in glaucoma is focused on lowering IOP by pharmacological means, surgically, or with laser treatment. However, patients continue to lose vision despite successful IOP control, and it is becoming clear that the exclusive management of IOP is not sufficient, and neuroprotection of retinal ganglion cells has been proposed as a potential alternative therapy [145].

Several studies have reported that the progressive degeneration of optic nerve axons and retinal ganglion cells in glaucoma is accompanied by chronic alterations in structural and functional characteristics of glial cells in the optic nerve head and retina [146, 147], where an abnormal microglial reactivity and redistribution take place [148]. TNF, IL-6, and IL-18 levels are increased in the retina and optic nerve head in both glaucomatous patients and animal models of glaucoma [149–151] and recent studies demonstrate that microglial activation is an early event in experimental models of glaucoma, which coincides with the onset of RGC death, potentially contributing to disease onset and/or progression [152–154]. Also, the treatment with minocycline, a tetracycline derivative known to reduce microglial activation [155], was able to improve retinal ganglion cell axonal transport and integrity in a mouse model of glaucoma [156].

### 1.8. Diabetic Retinopathy: A Low-Grade Inflammatory Disease

Diabetic retinopathy is one of the most common complications of diabetes and the most frequent cause of new cases of blindness among adults aged 20–74 years. After 20 years of diabetes, nearly all patients with type 1 and more than 60% of patients with type 2 diabetes have some degree of retinopathy [157]. Diabetic retinopathy has been considered a microvascular disease, but growing evidence demonstrates that retinal neurodegeneration also occurs [158–160], and diabetic retinopathy is now more accurately defined as a neurovascular disease.

Diabetic retinopathy exhibits characteristics of a chronic inflammatory process: increased levels of cytokines, such as IL-1*β*, IL-6, and TNF, have been found in the vitreous fluid of diabetic patients [161–163]; retinal TNF levels are also increased in diabetic patients, particularly in those with proliferative diabetic retinopathy [164–166]. The inflammatory profile of diabetic retinopathy has been confirmed in animal models of diabetes, where an increase was found in the levels of IL-1*β* [167–170] and TNF [170–172] in the retina. Therefore, the role of inflammation is unequivocal in diabetic retinopathy, from the leukocyte adhesion [173, 174] to the increase in inflammatory mediators, such as TNF, which exerts a crucial role in blood retinal barrier breakdown [175], as well as the death of retinal neurons [176]. As occurs in neurodegenerative brain diseases, microglial activation in the retina is also present in different stages of human diabetic retinopathy [177] and further reported in animals models of type 1 [170, 178–180] and type 2 [181] diabetes.

### 1.9. Is There a Role for A_2A_R in Retinal Degenerative Diseases?

Retinal ischemia is a common cause of visual impairment and blindness (reviewed in [182]). Retinal degeneration after ischemia-reperfusion injury by transient elevation of IOP in rats exhibits an extensive damage at the level of the retinal ganglion cell layer [183], similarly to that reported in human glaucoma [184]. Therefore, IOP-induced retinal ischemia has been extensively used as an animal model of acute glaucoma [185], in which activation of microglia has also been observed [36]. The role of A_2A_R in retinal ischemia-reperfusion injury is still controversial. On one hand, the treatment with a selective A_2A_R antagonist protects retinal function and structure in a model of retinal ischemia [186, 187]. On the other hand, it was reported that administration of an A_2A_R agonist prevents retinal thinning induced by ischemia-reperfusion damage [188].

Traumatic optic neuropathy is an important cause of severe vision loss in 0.5 to 5% of patients with closed head trauma [189]. Trauma is known to cause immediate mechanical damage to the axons of retinal ganglion cells, leading to degeneration. The death of retinal ganglion cells after optic nerve damage seems to be related to the local production of ROS and inflammatory mediators from activated microglial cells [190]. Increased phagocytic and proliferative microglia have been reported after optic nerve injury [191–193]. In the optic nerve crush injury mouse model, an important experimental disease model for traumatic optic neuropathy, a selective A_2A_R agonist decreased microglial activation, retinal cell death, and release of ROS and proinflammatory cytokines [190]. Moreover, levels of TNF and Iba-1 (a marker of cells from the myeloid lineage, including microglia) are increased in A_2A_R-knockout mice with optic nerve crush. In a different model of retinal degeneration, diabetic retinopathy, it was recently shown that A_2A_R mRNA transcripts and protein levels increase in the retina of type 1 diabetes models and also in retinal cell cultures exposed to elevated glucose concentration, used to mimic hyperglycemic conditions [194, 195]. A_2A_R-knockout diabetic mice exhibit increased cell death and TNF levels as compared with diabetic wild-type mice [179]. Accordingly, the administration of a selective A_2A_R agonist resulted in opposite effects upon cell death and TNF levels [179].

Experiments performed* in vitro* emphasize the controversial role played by A_2A_R in the control of retinal neuroinflammation. While some authors reported that the activation of A_2A_R attenuates LPS-induced release of TNF in retinal microglia [190], others found that A_2A_R blockade prevents LPS-induced increase in NO [196]. Moreover, A_2A_R blockade inhibits the LPS-induced increase in TNF expression and phagocytosis. In a more complex system, the retinal organotypic culture, A_2A_R blockade inhibits the expression of inducible NO synthase [196].

In summary, it remains to be clarified whether A_2A_R activation or blockade is the best approach to pharmacologically control neuroinflammation in the retina. This dual neuroprotective ability of A_2A_R modulation seems to be related with the specific inflammatory profile of different pathologies or pathologic conditions, as well as with the temporal window of neuroinflammation where the exposure to A_2A_R agonists or antagonists occurs. Although the controversy exists, most studies in brain pathology point towards a neuroprotective effect of A_2A_R blockade, in line with the ability of selective and nonselective A_2A_R antagonists to decrease most microglial functions.

## 2. Concluding Remarks

**Figure 4:**
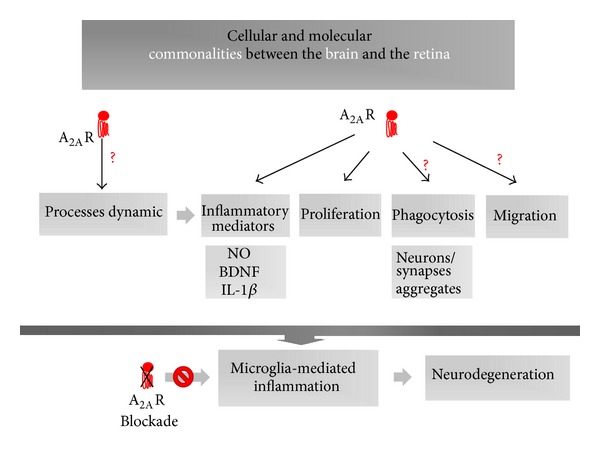
Cellular and molecular commonalities between the brain and the retina. Scheme identifying main microglial functions under the control of A_2A_R: release of inflammatory mediators and cellular proliferation. It remains to clarify if process extension/retraction (which supports the homeostatic surveying role of microglia), phagocytosis, and cellular migration are directly regulated by A_2A_R modulation (question marks). A_2A_R modulation is proposed as a promising pharmacological tool in the control of the chronic inflammatory process underlying degenerative conditions of the retina, based on similarities with microglia-mediated inflammation in brain disorders.

Brain degenerative diseases, such as AD and PD, are associated with microglial activation and chronic neuroinflammation. In both pathologies, the blockade of A_2A_R emerges as a candidate mechanism of neuroprotection, through the control of microglial reactivity. Glaucoma and diabetic retinopathy are retinal degenerative diseases, in which neuroinflammation also plays a crucial role. In the retina, microglial cells are also equipped with A_2A_R. Therefore, it is plausible to assume that A_2A_R modulation may also have a potential protective effect upon inflammation underlying degenerative processes of the retina (Figure 4). It remains to be clarified whether A_2A_R modulation has a net positive effect in the control of clinical features and progression of retinal degenerative diseases.
